# Incidence of Delirium in ICU Patients With and Without COVID-19 in a Costa Rican Hospital

**DOI:** 10.7759/cureus.70007

**Published:** 2024-09-23

**Authors:** Esteban Zavaleta-Monestel, Ernesto Martínez-Vargas, José Chaverri-Fernández, José Pablo Díaz-Madriz, Abigail Fallas-Mora, Paola Alvarado-Ajun, Carolina Rojas-Chinchilla, Jonathan García-Montero

**Affiliations:** 1 Pharmacy, Hospital Clínica Bíblica, San José, CRI; 2 College of Medicine, Universidad de Costa Rica, San José, CRI; 3 Pharmacology, Toxicology and Drug Dependence, Universidad de Costa Rica, San José, CRI; 4 Nursing, Hospital Clínica Bíblica, San José, CRI

**Keywords:** covid-19, delirium, intensive care unit, mechanical ventilation, sedation

## Abstract

Introduction: Delirium is a common and serious neurological complication in intensive care units (ICUs), often leading to poor patient outcomes and increased mortality. This study aimed to compare the incidence of delirium in ICU patients with COVID-19 to those with other respiratory infections in a private hospital in Costa Rica. Additionally, it evaluated the prevalence, severity, duration, and treatment of delirium in these critically ill patients.

Methods: A retrospective observational study was conducted, analyzing multiple variables obtained from the electronic health records of patients hospitalized in the ICU of Hospital Clinica Biblica. The study included patients admitted between January 2020 and December 2023. It compared the incidence of delirium among patients admitted for COVID-19 and those admitted for other diagnoses. The main outcomes measured were the incidence of delirium and the correlation of its management with international guidelines. The measures included the use of mechanical ventilation, the development of delirium, and the use of sedatives.

Results: A total of 137 patients were analyzed, of whom 57.7% were over 70 years old, 67.2% were men, 45.2% were admitted with a diagnosis of COVID-19, 90.5% used mechanical ventilation, and 49.6% of patients developed delirium. Dexmedetomidine was the most used sedative, which was the only one that showed a significant relationship with the development of delirium (p=0.0002). Delirium management was mainly done through the administration of dexmedetomidine (52.9%) and quetiapine (41.2%). There was no correlation between delirium development and mortality (p=0.2670).

Conclusion: The study results do not show a significant relationship between COVID-19-positive patients and the development of delirium. Similarly, no higher mortality was observed in those patients who experienced delirium during their ICU stay.

## Introduction

Delirium is a serious neurological complication that can develop in hospitalized patients over a short period, ranging from hours to days. It is associated with a variety of complications in hospitalized patients, including those diagnosed with COVID-19 [1-3]. Currently, the specific incidence of delirium in COVID-19 patients admitted to intensive care units (ICUs) is not fully understood. However, it has been established that these individuals have a heightened risk of developing delirium [4]. In patients without this diagnosis, the relative incidence of delirium can be up to 30%, which significantly increases to 80% among patients treated in the ICU [5].

Historically, delirium within the hospital setting has been associated with poor outcomes in medical evolution, with increased mortality being the most relevant consequence. Although this syndrome may be transient and reversible, it is associated with an increased incidence of subsequent dementia after discharge from the ICU [6].

Tools such as the Confusion Assessment Method for ICU (CAM-ICU) or the Intensive Care Delirium Screening Checklist (ICDSC) allow for the rapid and straightforward identification of delirium using variable scales. However, delirium screening in hospitalized patients is an uncommon practice within healthcare services. This issue was exacerbated during the peak years of the COVID-19 pandemic due to factors such as patient prioritization and isolation, incorporation of untrained personnel into the healthcare team, and increased workload burden [7].

Therefore, the prevalence of delirium in patients diagnosed with COVID-19 is likely higher than reported, emphasizing the potential long-term consequences, and highlighting the need to understand the incidence of this condition [7]. Prevention, early detection, and management of modifiable factors in the hospital environment to minimize the risk and effects of delirium in ICU patients are fundamental to improving clinical outcomes [8,9].

Currently, there is an urgent need for studies that provide information to mitigate risk factors and enable healthcare professionals to better understand the underlying pathology of delirium in ICU patients. This understanding will aid in more effectively identifying possible additional treatments and therapies. Triggering factors, incidence, prevention, drugs, and rehabilitation for patients with delirium are critical areas for study to improve short-and long-term outcomes for patients with this condition in the ICU [10].

Objective

The aim of this study was to compare the development of delirium in patients with COVID-19 and those with other respiratory infections admitted to the ICU of a private hospital in Costa Rica. In addition, the study sought to analyze the prevalence, severity, duration, and treatment of delirium in these critically ill patients.

## Materials and methods

Study design and settings

A retrospective observational study was conducted involving patients admitted to the ICU of Hospital Clínica Bíblica from January 2020 to December 2023. The study included two patient groups: the first group comprised patients with a positive COVID-19 test result confirmed either via polymerase chain reaction (PCR) or SARS-CoV-2 antigen testing, while the second group consisted of patients with diagnoses warranting ICU admission unrelated to COVID-19.

Inclusion and exclusion criteria

Inclusion criteria required patients to be over 18 years old without pre-existing neurological disorders or delirium episodes prior to ICU admission, with a stay in the ICU of more than 24 hours and sedation during their stay. Pregnant women and patients with incomplete clinical records were excluded.

Data collection

The study identified patients hospitalized during the specified periods by consulting the hospital's electronic medical records. A data collection sheet was created to gather general patient characteristics, including age, gender, length of hospital stay, duration of ICU stay, need for mechanical ventilation, associated comorbidities and sedative medication used.

The description of the mental status and the evolution of delirium were derived by analyzing the following internal information and symptomatology reported in the electronic medical records: disorientation, asynchrony with the ventilator, inability to follow simple commands or agitation, blank stares, verbosity, need for restraints, and removal of catheters or peripheral lines by the patient.

Statistical analysis

The statistical analysis was conducted using RStudio software, version 4.3.0 (The R Foundation for Statistical Computing, Vienna, Austria). A Z-test for two proportions was employed to identify significant differences in the presence of certain comorbidities between patients who developed delirium and those who did not. This test was also crucial in determining if there is a significant relationship between delirium and variables such as age, gender, mortality, the use of mechanical ventilation, or the reason for admission to the ICU. Additionally, it was used to compare medication usage between COVID-19 and non-COVID-19 patient groups.

Ethics approval and consent to participate

The study was reviewed by the Scientific Ethical Committee of the University of Costa Rica Board (IRB approval number CEC-554-2023, amendment approved on September 25, 2023) and was granted exemption status from patient consent due to its retrospective, non-interventional design. All procedures were conducted in accordance with the ethical standards of the responsible committee on human experimentation and the Helsinki Declaration of 1975.

## Results

**Figure 1:**
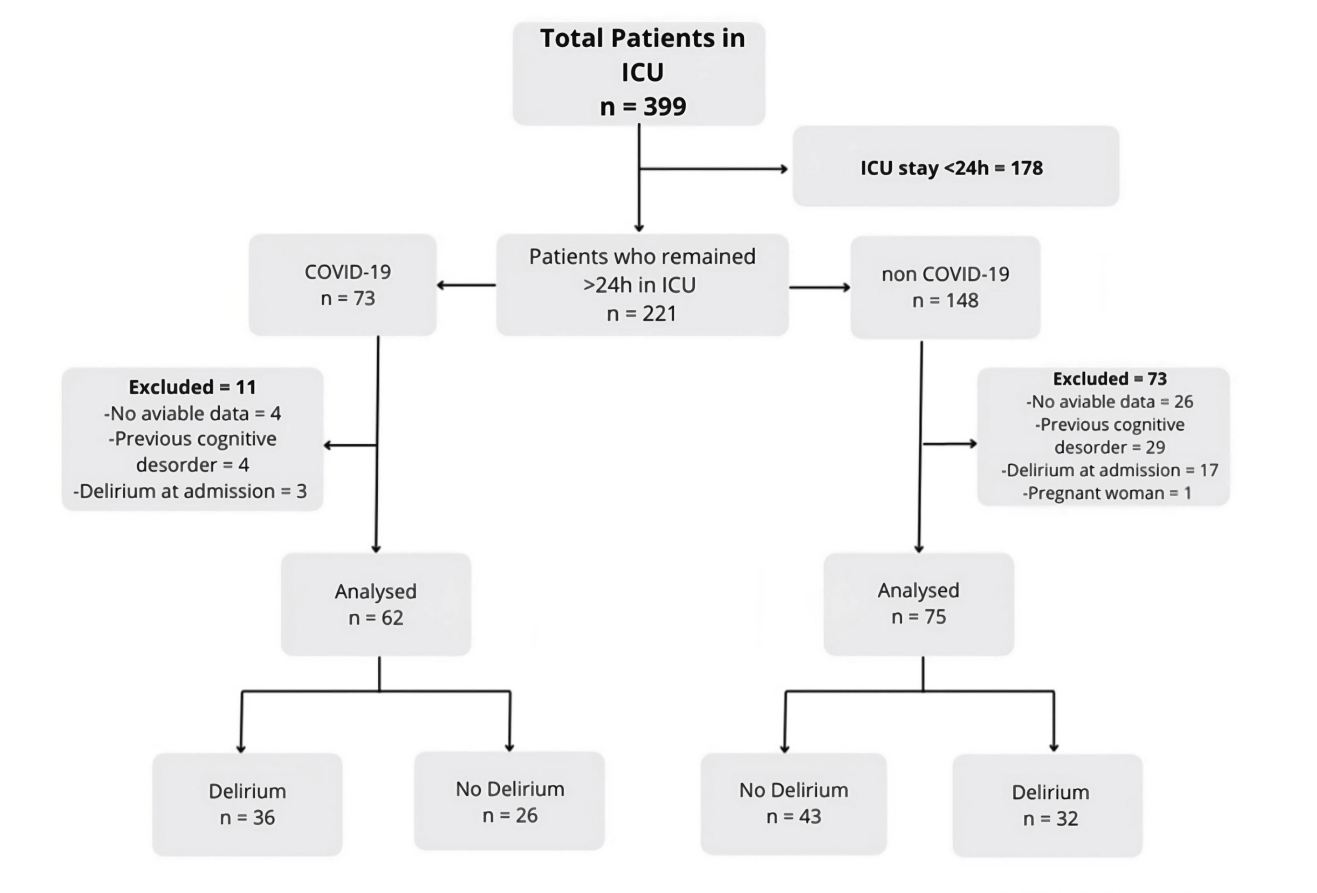
Selection and stratification criteria for the study population

Out of the 399 patients analyzed, only 221 met the inclusion criteria of an ICU stay of more than 24 hours. Among the 221 patients in this category, 73 were admitted with a diagnosis of COVID-19, while 148 presented with other medical conditions at the time of admission. Figure 1 shows the patient selection and exclusion process for both the COVID-19 group and the non-COVID-19 group in this study.

After applying the remaining exclusion criteria, the final analysis included a final group of 137 patients: 62 in the COVID-19 group and 75 in the non-COVID-19 group. The age distribution ranged from 30 to 97 years, with 57.7% (n=79) of patients aged over 70, among whom the highest incidence of delirium (51.5%) was observed. Regarding gender, 67.2% (n=92) were male. Table 1 provides detailed demographic information, including age and gender distribution, along with delirium incidence.

**Table 1:** Proportion of patients by age and gender and incidence of delirium development

Variable	Patients, n (%)	Incidence of delirium, %
Age		
30-50	10 (7.3)	10.3
51-70	48 (35)	38.2
>70	79 (57.7)	51.5
Gender		
Female	45 (32.8)	30.9
Male	92 (67.2)	69.1

The length of hospital stay ranged from two to 51 days for the non-COVID-19 group, the group of two to 10 hospitalization days had the largest number of patients. In contrast, for the COVID-19 group hospitalization time ranged from two to 61 days, with most of the patients (40.3%, n=25) staying for 11 to 20 days. Regarding ICU stay, in the COVID-19 group, the number of days ranged between two and 51 days, with 81.3% (n=61) of the patients in the group of two to 10 days of hospitalization. In contrast, patients with COVID-19 had a maximum ICU stay of 30 days, with 59.7% (n=37) staying for two to 10 days (Table 2).

**Table 2:** Length of hospital stay and ICU stay

Days	Length of hospital stay for COVID-19 group (n=62), n (%)	Length of hospital stay for non-COVID-19 group (n=75), n (%)	Length of ICU stay for COVID-19 group (n=62), n (%)	Length of ICU stay for non-COVID-19 group (n=75), n (%)
2-10	23 (37.1)	52 (69.3)	37 (59.7)	61 (81.3)
11-20	25 (40.3)	12 (16.0)	19 (30.6)	8 (10.7)
More than 20	14 (22.6)	11 (14.7)	6 (9.7)	6 (8.0)

In the non-COVID-19 group (n=75), the most observed comorbidities were hypertension (74.67%, n=56), diabetes (26%, n=19), dyslipidemia (28%,n=21), heart failure (16%, n=12), arrhythmias (19%, n=14), and renal insufficiency (32%, n=24). Conversely, in the COVID-19 group (n=62), hypertension was observed in 51.61% of patients (n=32), diabetes in 20.97% (n=13), dyslipidemia in 24.19% (n=15), heart failure in 1.61% (n=1), arrhythmias in 11.29% (n=7), and renal insufficiency in 3.23% (n=2). As shown in Table 3, none of these comorbidities had a significant relationship with the development of delirium.

**Table 3:** Delirium development in the study population *Other: Aortic valve replacement, treatment of renal insufficiency, knee bursa resection, ureteroscopy. The result is considered significant if the p-value is less than 0.05.

Baseline characteristic	Patients with delirium (n=68), n (%)	Patients without delirium (n=69), n (%)	p-value
Age >70 years	35 (51.5)	44 (63.8)	0.1443
Male gender	47 (69.1)	45 (68.2)	0.6241
Reason for ICU admission			
Cardiovascular	8 (11.8)	12 (17.4)	0.3524
Gastrointestinal	3 (4.4)	9 (13.0)	0.0735
Metabolic	2 (2.9)	1 (1.4)	0.5485
Infection	7 (10.3)	2 (2.9)	0.0801
Trauma	7 (10.3)	11 (15.9)	0.3271
Respiratory (no COVID)	2 (2.9)	7 (10.1)	0.0891
COVID-19	36 (52.9)	26 (37.7)	0.0735
Other*	3 (4.4)	1 (1.4)	0.3030
Comorbidities			
Hypertension	44 (64.71)	44 (63.8)	0.9124
Diabetes Mellitus	19 (27.9)	20 (29.0)	0.8887
Dyslipidemia	21 (30.9)	22 (31.9)	0.8966
Heart failure	5 (7.4)	8 (11.6)	0.3953
Arrhythmia	12 (17.6)	14 (20.3)	0.6966
Renal insufficiency	14 (20.6)	12 (17.4)	0.6312
Medications			
Midazolam	56 (82.6)	48 (69.6)	0.0801
Propofol	43 (63.2)	35 (50.72)	0.1389
Opioids (Fentanyl)	22 (32.4)	20 (29.0)	0.6672
Ketamine	1 (1.47)	0 (0.0)	-
Dexmedetomidine	58 (85.3)	39 (56.5)	0.0002
Neuromuscular blockers	34 (50.0)	20 (29.0)	0.0117
Use of mechanical ventilation	64 (94.1)	60 (87.0)	0.1527
Mortality	31 (45.6)	38 (55.1)	0.2670

The relationship between COVID-19 infection and the development of delirium was not statistically significant (p = 0.0740). Overall, 49.64% (n=68) of the patients developed at least one episode of delirium, with an incidence of 58.1% (n=36) in the COVID-19 group and 42.7% (n=32) in the non-COVID-19 group (Table 3).

A higher percentage of patients in the COVID-19 group required mechanical ventilation (93.5%, n=58), with an average duration of 8.68 days (Table 3). Of these patients, 60.3% (n=35) developed delirium. In the non-COVID-19 group, 88% (n=66) required mechanical ventilation, with an average duration of 5.97 days, and 29 of these patients (43.9%) developed delirium. There was no significant difference in the effect of mechanical ventilation on the development of delirium between COVID-19 and non-COVID-19 patients (p = 0.4460).

There was no significant relationship between the development of delirium and mortality (p = 0.2670). Of the patients who developed delirium, 45.6% (n=31) died, compared to 55.1% (n=38) of the patients who died without developing delirium (Table 3).

**Figure 2:**
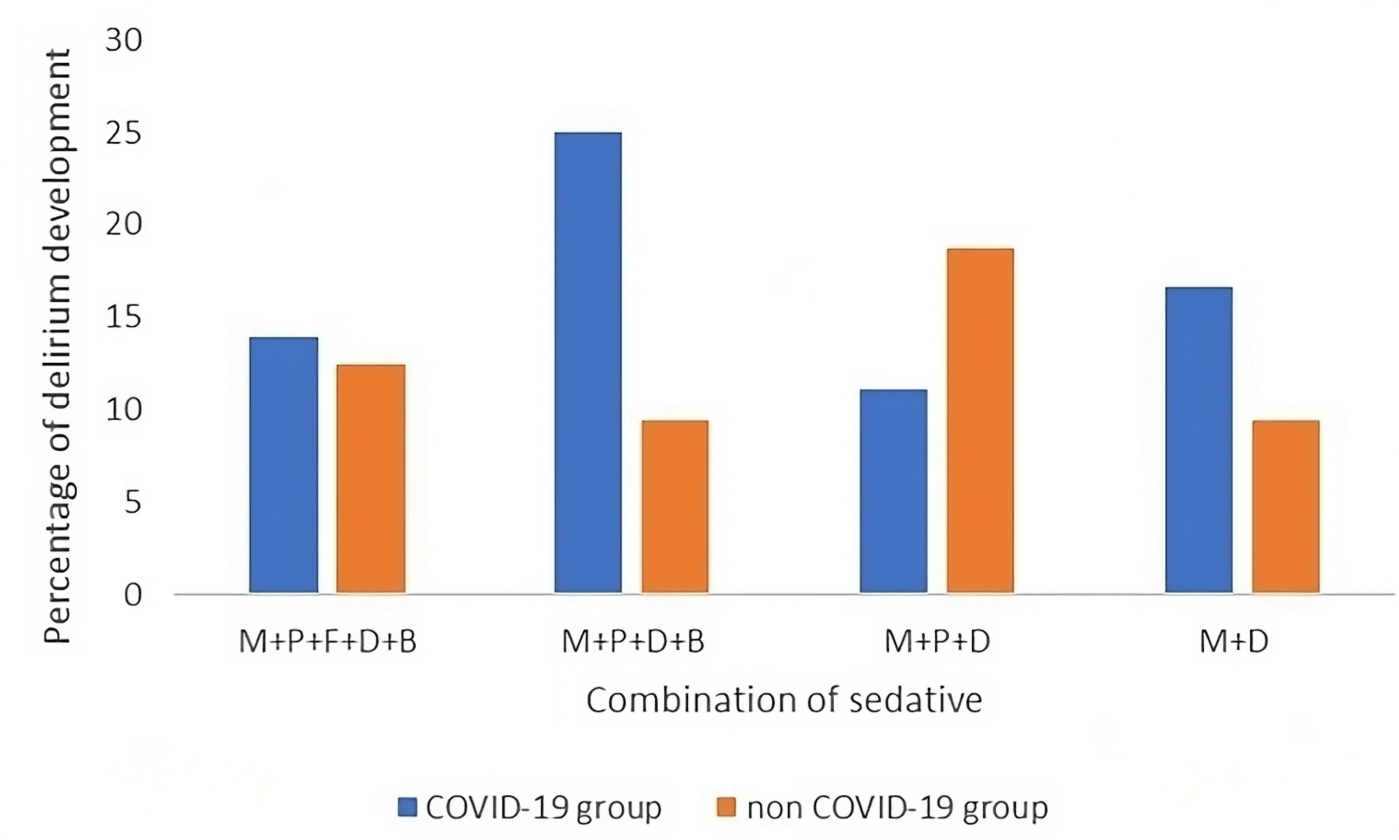
Sedative combinations with highest delirium rates M: midazolam; P: propofol; F: fentanyl; D: dexmedetomidine; B: neuromuscular blockers

Furthermore, there was a significant relationship between the duration of sedative use and the development of delirium (p = 0.0285). Figure 2 displays the four drug combinations most associated with delirium in both study groups. It is important to highlight that all these combinations include dexmedetomidine.

A significantly higher use of sedative drugs, opioids, and neuromuscular blockers was observed in ICU patients with COVID-19, who had an average sedation period of 8.16 days, compared to 5.37 days in those admitted for other medical conditions. As seen in Table 4, the increased utilization of dexmedetomidine and neuromuscular blockers was noteworthy among COVID-19 patients.

**Table 4:** Comparison of drug usage in COVID-19 and non-COVID-19 patients in the ICU and management of delirium The result is considered significant if the p-value is less than 0.05.

Drug	COVID-19 group, n (%)	Non-COVID-19 group, n (%)	p-value
Usage in ICU			
Midazolam	45 (72.6)	59 (78.7)	0.407
Propofol	34 (54.8)	44 (58.7)	0.653
Fentanyl	14 (22.6)	28 (37.3)	0.063
Ketamine	1 (1.6)	0 (0.0)	-
Dexmedetomidine	55 (88.7)	42 (56.0)	< 0.001
Neuromuscular blockers	32 (51.6)	22 (29.3)	0.008
Usage for delirium			
Quetiapine	16 (44.4)	12 (37.5)	0.0761
Olanzapine	1 (2.8)	1 (3.1)	0.386
Dexmedetomidine	19 (52.8)	17 (53.1)	0.592
Propofol	0 (0.0)	2 (6.3)	-

A total of 12 patients received quetiapine as a preventive treatment for delirium (two from the non-COVID-19 group and 10 from the COVID-19 group), of which one patient (8.3%) in the COVID-19 group developed delirium. Table 4 outlines the pharmacological management of patients who develop delirium.

Among the 28 patients who received quetiapine as a treatment for delirium, only seven (25.0%) had the medication discontinued once the delirium was resolved. The remaining 21 patients (75.0%) continued receiving quetiapine even after their delirium was resolved (Table 4).

Conversely, all patients treated with olanzapine or propofol had their medications discontinued once the delirium was resolved. However, patients who received dexmedetomidine continued the drug administration even after their delirium had subsided. Overall, 11 out of 68 patients (16.2%) discontinued treatment for delirium once the condition was resolved. The remaining 57 patients (83.8%) continued treatment after the resolution of delirium (Table 4).

## Discussion

Previous reports have suggested a possible association between a confirmed COVID-19 diagnosis and the development of delirium. It is estimated that 20-30% of hospitalized patients with COVID-19 experience delirium, with this figure reaching up to 70% in critically ill patients [11-14]. Delirium is a critical, systemic, and infectious disorder, influenced by a series of predisposing factors including respiratory failure, increased need for mechanical ventilation, prolonged use of sedative agents, and extended hospital stays under strict isolation measures [1-8,15,16].

However, the results obtained in this study, as shown in Table 3, did not reveal a statistically significant relationship between the development of delirium and COVID-19 infection. These findings should be interpreted with caution, as other factors may affect the presence or absence of delirium and its proper detection in critically ill patients [7].

In the hospital under study, the detection of delirium in hospitalized patients is infrequent. The determination of delirium was based on observed symptoms rather than the use of standardized tools such as the CAM-ICU or the ICDSC. These tools facilitate rapid and systematic identification of delirium through different scales and are supported by recognized international guidelines, including the Clinical Practice Guidelines for the Prevention and Management of Pain, Agitation/Sedation, Delirium, Immobility, and Sleep Disruption in Adult Patients in the ICU (PADIS) [7,17,18].

Additionally, during the peak years of the COVID-19 pandemic, accurate identification of delirium by healthcare professionals was hindered due to prioritization and isolation of patients, incorporation of untrained personnel into the healthcare team, workload overload, and the use of personal protective equipment [7]. The lack of systematic recognition of delirium symptoms and the variability in the diagnostic criteria suggest that the prevalence of delirium in patients diagnosed with COVID-19 has likely been underestimated in the clinical records available to date. Other factors predisposing individuals to the development of delirium include psychiatric history and advanced age [4,19,20]. However, individuals with pre-existing neurological disorders were excluded from this study.

Previous studies indicate a higher risk and prevalence of delirium in elderly patients, with rates ranging from 8% to 17% among those admitted to emergency departments and up to 40% in nursing homes [21,22]. The results of this study similarly show that the oldest age group had the highest incidence of delirium (Table 1). However, a significant relationship between age and delirium could not be established (Table 3).

With respect to gender, a higher incidence of delirium was observed in male patients. Although our results do not support a significant association between gender and the development of delirium, previous literature does confirm a significant relationship, particularly noting that male patients often present with symptoms like agitation, hallucinations, impulsivity, and combativeness more frequently than female patients [23].

The presence of certain comorbidities in critically ill patients can increase the risk of developing delirium. However, for conditions such as hypertension, diabetes, or renal failure to contribute to the onset of delirium in ICU patients, they typically need to be poorly controlled for an extended period [24-26]. Consequently, establishing a direct link between these comorbidities and delirium in this study is challenging. As shown in Table 3, no significant relationship was found between these conditions and the incidence of delirium in patients, despite their presence in both groups.

The use or non-use of mechanical ventilation is a key factor in the development of delirium in critically ill patients [2,5,7,14,27,28]. Previous studies indicate that mechanical ventilation can increase the risk of delirium by up to 80% in ICU patients [29]. However, our results do not align with these findings. As shown in Table 3, no significant relationship was found between mechanical ventilation and the development of delirium. Both groups had a similar number of patients who developed delirium and those who did not while under mechanical ventilation.

The use of sedatives is another factor that can increase the incidence of delirium. Midazolam is commonly used as a sedative drug in the ICU due to its limited effect on hemodynamics and short half-life, but previous studies show that the use of benzodiazepines such as midazolam is often associated with the onset of delirium [30,31].

Dexmedetomidine stands as one of the most frequently used drugs for sedation in ICU patients, offering some advantages over midazolam, such as its non-suppressive effect on the respiratory system during sedation and lower incidence of delirium development [32,33]. Despite previous studies endorsing dexmedetomidine for hastening awakenings compared to midazolam and consequently lowering the risk of delirium [31], our findings diverged. In this study, patients receiving dexmedetomidine exhibited a higher rate of delirium development compared to those administered midazolam, propofol, ketamine, fentanyl, or neuromuscular blockers (Table 3). Notably, a statistically significant relationship emerged between dexmedetomidine usage and delirium development. However, it’s worth noting that many patients were concurrently administered several of these medications.

The predominance of dexmedetomidine-midazolam combinations among the most frequently prescribed drug combinations, as depicted in (Figure 2), underscores a noteworthy practice trend. However, the use of dexmedetomidine at high doses and concurrently with other sedative drugs or neuromuscular blockers may exacerbate the risk of delirium and adverse effects. This combined approach amplifies sedative effects, CNS depressants, and other undesirable side effects, including delirium [27]. Furthermore, recent research has failed to establish an association between dexmedetomidine and reduced delirium risk compared to other sedatives [34]. This observation could explain the significant association found between neuromuscular blocker use and delirium (Table 3). While factors like immobility and increased sedation depth may contribute [35], no prior studies definitively support their correlation with delirium development.

Regarding delirium management, patients were treated preventively solely with the use of quetiapine. Currently, the use of antipsychotics for delirium prevention remains controversial, with varied study results [36-41]. However, international guidelines such as the Diagnostic and Statistical Manual of Mental Disorders, Fifth Edition (DSM-5) recommend employing low doses of antipsychotics for a limited duration as a preventive measure against delirium development in the absence of evidence confirming their efficacy [27,42-44].

For patients presenting delirium, as outlined in Table 3, dexmedetomidine was the primary drug administered in both groups. Additionally, a notable usage of quetiapine and, to a lesser extent, olanzapine was observed. While dexmedetomidine isn't the first-line treatment, it has demonstrated benefits in alleviating delirium-associated agitation, primarily by reducing sympathetic flow from the CNS and indirectly minimizing the need for other potentially delirium-inducing agents. It's imperative to cease dexmedetomidine use once the delirium event resolves [45-48]. However, as previously discussed, concurrent dexmedetomidine use with other sedative agents may contribute to the observed association between its use and delirium development.

Antipsychotics remain the primary pharmacological treatment for delirium, although their efficacy for this purpose remains controversial [49,50]. Nonetheless, studies suggest their benefits outweigh the risks when employed to manage specific symptoms like agitation, paranoia, or psychosis, provided the treatment is limited to delirium resolution and not needlessly extended [46,48,51]. Although propofol usage for delirium treatment was minimal in this study, it's not included in the recommended medications for delirium management. Its use for this purpose lacks support from delirium treatment guidelines or previous research [44,48].

Moreover, the most frequently used medications for managing delirium in this study, dexmedetomidine and quetiapine, have demonstrated efficacy and are endorsed by guidelines such as the DSM-5 and previous studies, albeit with the aforementioned limitations [44,48]. Nonetheless, delirium management fell short due to failure to discontinue drug administration upon delirium resolution, leading to prolonged usage in most treated patients [27,44,48].

Contrary to previous research identifying delirium as a determinant for increased short-term mortality risk, no direct relationship between delirium and mortality was observed in this study. Understanding the complex interplay between delirium and mortality underscores the importance of a multidisciplinary approach to its understanding and treatment [52-54].

Limitations

A principal limitation of this study is its retrospective design, which constrains the scope of the analysis to data that has already been collected. This may have introduced potential distortions and inaccuracies in the findings. The utilization of historical data may entail the presence of inherent biases, such as the exclusion of crucial information or inconsistencies in the documentation of clinical records. Additionally, the limited sample size of patients in both groups was constrained by the 75-bed capacity of our private hospital. It is acknowledged that the findings of this study cannot be extrapolated to a national level or applied to other hospital centres. However, the value of this research lies in its provision of relevant local data, which can serve as a foundation for future multicenter studies.

A further limitation is the lack of detailed information in the electronic records regarding the presence of delirium or associated symptoms. This is partially attributable to the absence of systematic documentation of suspicious signs and symptoms, as well as the dearth of formal evaluations conducted using validated tools for the detection of delirium. The lack of these data precludes a comprehensive evaluation of the issue.

To address these limitations in future studies, we propose the implementation of a clinical guideline based on the most recent evidence regarding the prevention and management of delirium in hospitalized patients. It is recommended that this guideline be accompanied by ongoing educational programs for healthcare professionals, with a focus on the accurate identification and documentation of delirium and its associated symptoms through the use of standardized assessment instruments. Such initiatives would not only enhance the quality of care but would also facilitate the availability of clinical data for future studies.

## Conclusions

This study found no significant difference in delirium incidence between ICU patients diagnosed with COVID-19 and those admitted for other reasons. Furthermore, no direct correlation was observed between delirium development and mechanical ventilation use or patient mortality. However, a higher incidence of delirium was noted in patients who received dexmedetomidine as a sedative drug and neuromuscular blockers. These findings emphasize the ongoing need for research into delirium triggers, prevention, detection, and management to enhance both short-term and long-term patient outcomes.
